# Complementary Feeding Methods: Associations with Feeding and Emotional Responsiveness

**DOI:** 10.3390/children10030464

**Published:** 2023-02-26

**Authors:** Carla Fernandes, Fátima Martins, Ana F. Santos, Marília Fernandes, Manuela Veríssimo

**Affiliations:** 1 William James Center Research, ISPA-Instituto Universitário, 1149-041 Lisbon, Portugal; 2 ISPA-Instituto Universitário, 1149-041 Lisbon, Portugal

**Keywords:** complementary feeding, baby-led weaning, weaning, feeding practices, emotion regulation, parental responsiveness

## Abstract

Learning to eat complementary foods is a crucial milestone for infants, having implications across development. The most used method for introducing complementary foods is Traditional Spoon-Feeding (TSF). However, the alternative method Baby-Led Weaning (BLW) is increasingly becoming used as it has been associated with positive outcomes. Research analyzing associations between complementary feeding methods and responsive parenting is practically non-existent. Therefore, the objective of this study was to analyze differences in emotional and feeding responsiveness between caregivers who previously implemented traditional vs. non-traditional feeding approaches. Caregivers (mostly mothers) of 179 children between 3 and 5 years were asked about the complementary feeding method that they had followed previously (70.4% reported using the TSF, 16.8% said they used the BLW and 12.8% used both methods simultaneously). In addition, they reported on their feeding practices using the Comprehensive Feeding Practices Questionnaire and on their responses to children’s distress using the Coping with Children’s Negative Emotions Scale. The results showed that parents who reported using a non-traditional (BLW or both) complementary feeding method reported less pressure to eat and minimization of reactions to children’s negative emotions, compared to parents who used a traditional method (although these reported using more problem-focused reactions). The findings suggest that complementary feeding methods and responsive parenting may be linked, leaving the question of which one sets the stage for the other.

## 1. Introduction

It is during early childhood that food habits start to develop, making this a crucial moment to promote healthy food choices. The change from breast milk or formula to complementary foods is a crucial developmental milestone and is related with eating behaviors, food preferences and body weight across development [1,2,3,4,5].

### 1.1. Complementary Feeding Methods

The WHO’s most recommended complementary feeding method is Traditional Spoon-Feeding (TSF) [6,7]. In this method, infants are spoon-fed by caregivers, and the first solids offered are pureed foods, with gradual exposure to more varied textures and flavors over time, until family foods are introduced [8,9].

An alternative method named Baby-Led Weaning (BLW) has become increasingly widely used. According to Rapley [10], this method allows the infant to lead the weaning process, choosing what, when and how fast to eat. Within this approach, family foods are offered to the infant, in a texture and form that are adapted to the child’s developmental stage, for example, in the form of pieces (finger foods) that he/she can grasp with their hands and eat independently [11,12]. Thus, infants can experience and participate in family meals [10,13,14]. Occasionally, spoon-feeding or serving pureed foods may occur for up to 10% of the total feeding time [1]. However, not all authors agree with this definition of BLW, suggesting that this method implies the infant self-feeds all or most of the time [15,16]. Although previous studies are limited in number and most of them are correlational, BLW has been associated with positive outcomes such as lower body mass index [17], preference for healthy foods [5], increased satiety responsiveness [1], a longer period of exclusive breastfeeding [8,15,18,19], children’s more frequent participation in family meals and enjoyment at mealtimes [8,15,19,20], lower maternal anxiety [2,17,19] and infant irritability [18,20].

In sum, in the TSF method, the caregivers’ control appears to be high, as they guide with a spoon the amount, speed, type and consistency of food given to the infant [19,21]. By contrast, in the BLW method, caregivers present a variety of solid foods, and the infant takes an active role, being allowed to self-feed and choose the food and quantities to eat [10]. Evidence suggests that there are differences between parents and children who follow each of these methods. Still, research has not yet been able to keep up with the growing interest in BLW, with many questions still open or to be clarified [2]. At this level, one question of interest is related to parents’ responsiveness to the child [22].

### 1.2. Feeding and Emotional Responsiveness and Complementary Feeding

Responsive parenting is characterized by the caregiver’s ability to adequately identify the different signals the child transmits and, in turn, give a developmentally appropriate response, in an emotionally supportive way, not intrusive or controlling [23]. Similarly, in the context of responsive feeding, the caregiver can recognize and respond appropriately to the child’s internal appetite cues, whereas, in non-responsive feeding, caregivers use excessive controlling and coercive feeding practices, failing to respond to these cues [23,24,25]. As a result, non-responsive feeding practices can undermine the child’s self-regulation of energy intake, increasing the risk of overweight or obesity development [23,26,27,28,29]. On the other hand, caregivers’ responsive feeding practices promote children’s ability to regulate their internal hunger and satiety cues [23]. Similarly, as previously referred to, evidence shows that BLW is also associated with greater satiety responsiveness, which could result from the opportunity infants have to regulate the amount of food they eat as they are the ones who feed themselves in this complementary method [1,9]. So, like responsive feeding, the BLW method increases children’s attention to hunger and satiety cues. In this sense, it is possible that BLW could be somehow related to responsive feeding practices or consist of a form of responsive feeding. However, to our knowledge, only one study has examined this link. In this study, Brown and Lee [30] found that mothers who used BLW reported less nonresponsive practices, namely, pressure to eat and restriction of food, compared with mothers who followed a more conventional method. Furthermore, when caregivers doubt children’s ability to learn to self-feed and consume enough food or feel stressed or pressured for some reason, they could end up dominating the feeding situation, turning to non-responsive feeding practices [23] and not endorsing the BLW method. Thus, caregivers may resort to a complementary food method in which their control is higher, such as the TSF method.

In addition, emotions are strongly present in mealtimes [31]. In particular, the introduction of new flavors and consistencies can lead the child to demonstrate positive and negative emotions. Moreover, children seem to have a biological predisposition to be reluctant or refuse new foods [32]. Thus, this refusal could lead to tensions between caregivers and children, and mealtime may become stressful and prompt negative emotions. The way caregivers choose to deal with children’s emotions during feeding may impact both children’s emotion regulation and regulation of energy intake [31]. In fact, recent research shows that emotional responsiveness and feeding responsiveness are intertwined, with the use of unsupportive emotional responses (e.g., distress, punitive and minimization responses) being a risk factor for the use of non-responsive feeding practices (e.g., pressure to eat, restriction, food as a reward, and emotion regulation) [31,33,34,35,36]. Therefore, as emotional unresponsiveness could lead caregivers to exert excessive control in feeding, which, in turn, places children at risk for excessive weight gain [27], it is important to assess whether emotional responsiveness could have a role in complementary feeding. Evidence at this level is nonexistent or at least difficult to locate.

### 1.3. Current Study

A small group of previous studies have highlighted the importance of emotional responsiveness to feeding responsiveness and, therefore, to children’s self-regulation of energy intake [31,33,34,35,36]. Additionally, there is also evidence associating BLW to children’s self-regulation of energy intake [1,37]. However, there is a dearth of literature about the relationship between emotional and feeding responsiveness and complementary feeding methods. Therefore, the main aim of this study is to analyze the differences in emotional and feeding responsiveness between caregivers who previously implemented the BLW or TSF method with their children.

## 2. Materials and Methods

### 2.1. Participants

Participants were the caregivers (mostly mothers, 98.3%) of 179 children (104 boys, 75 girls), aged between 3 and 5 years old (*M* = 51.5 months; *SD* = 11.3) from Lisbon, Portugal. Most parents were either married or cohabitating (83.7%). Mother’s age ranged between 23 and 48 years (*M* = 36.7; *SD* = 5.1), 42.5% had a master’s degree (30.2% had high school diploma, 2.8% had a bachelor’s, 20.7% had an advanced professional degree and 1.7% a PhD) and most of them (78.8%) worked full-time (12.3% work part-time, and 8.4% were unemployed). Father’s age ranged between 22 and 58 years (*M* = 38.8; *SD* = 5.9), 53.1% had a high school diploma (0.6% bachelor, 27.4% master, 8.4% advanced professional degree and 4.5% and a PhD), most fathers (93.9%) work full-time (1.7% work part-time, and 2.2% were unemployed).

Children usually spend an average of 7 h at school (*SD* = 1.5), 53.7% were firstborn and 67% had siblings. Children’s weight varied between 10 kg and 32 kg (*M* = 17.7; *SD* = 3.7), and their height varied between 87 cm and 130 cm (*M* = 106.1; *SD* = 8.3). The beginning of the children’s food introduction ranged between 3 months and 8 months (*M* = 5.3; *SD* = 1.2).

### 2.2. Measures

#### 2.2.1. Complementary Feeding Methods

Caregivers were asked to retrospectively self-identify themselves as following a TSF, BLW or a mixed complementary feeding method. A definition of TSF and BLW was provided to caregivers. For parents that felt that they did not fit in either of these complementary feeding methods, an additional option was provided. In this, they were asked to describe the method they used. The mixed feeding method emerged after the analysis of caregivers’ responses corresponding to those who indicated to use a combination of both TSF and BLW methods. In order to double check this information, they were also asked to estimate the frequency of its use and the proportion of food that they provided as purées or spoon-fed to their children when they were babies. These notions are present among caregivers who perceive themselves as following a particular complementary feeding method, for example, BLW (e.g., [1,2,22]).

#### 2.2.2. Parental Feeding Practices

Caregivers’ feeding practices were assessed using the Comprehensive Feeding Practices Questionnaire (CFPQ) [38], a questionnaire composed of 49 items that are answered by caregivers using a 5-point rating scale, indicating their degree of agreement (1 = disagree, to 5 = agree; items 1–13) or their frequency of use a specific feeding approach (1 = never, to 5 = always; items 14–49). Items can be aggregated into 12 subscales reflecting distinct caregivers’ feeding practices. Six of these subscales reflect more positive or healthy feeding styles, namely: *encourage balance and variety* (α = 0.71), i.e., promoting of healthy and varied food consumption; *environment* (α = 0.63), i.e., providing healthy foods; *involvement* (α = 0.72), i.e., encouraging child’s involvement in food preparation and in meal planning; *modelling* (α = 0.63), i.e., being an active and enthusiastic model of healthy eating for the child; *monitoring* (α = 0.82), i.e., keeping track of child’s intake of unhealthy foods; and *teaching about nutrition* (α = 0.42), i.e., encouraging the child’s intake of healthy foods through didactic techniques. The other six subscales reflect unhealthy, emotion-related or pressuring feeding styles, including *emotion regulation* (α = 0.82), i.e., using food to regulate the child’s emotions; *child control* (α = 0.58), i.e., allowing the child to control their feeding interactions and own eating behaviors; *food as reward* (α = 0.51), i.e., using of food as a reward for child’s behavior; *pressure* (α = 0.70), i.e., encouraging the child to eat more food at meals, ignoring the child’s satiety/hunger cues; *restriction for weight* (α = 0.79), i.e., controlling the child’s intake to maintain or decrease the child’s weight; and *restriction for health* (α = 0.58), i.e., controlling the child’s intake to refers to parental control of child’s intake in order to limit unhealthy foods.

Following Bost and colleagues [33], and excluding subscales with α < 0.60, two composites were generated: the *pressuring feeding styles* (average of *emotion regulation*, and *pressure* α = 0.74) and the *healthy feeding styles* (average of *modeling, involvement*, *encourage balance and variety*, and *environment*; α = 0.71).

#### 2.2.3. Caregivers’ Responses to Children’s Negative Emotions

Caregivers’ responsiveness to children’s negative emotions was assessed using the Coping with Children’s Negative Emotions Scale (CCNES) [39]. It includes 12 hypothetical scenarios, each with 6 possible and qualitatively different parental reactions to the child when he/she is upset or expressing negative emotions (e.g., “If my child becomes angry because he/she is sick or hurt and can’t go to his/her friend’s birthday party, I would…”). For each one, parents should indicate how likely they are to react in those specific ways using a 7-point rating scale (1 = very unlikely; 7 = very likely). These distinct parental reactions to control or regulate the child’s negative emotional expression correspond to 6 subscales. Three of them reflect negative reactions: *punitive reactions* (α = 0.78), involving caregivers’ use of punishment (verbal/physical); *distress reactions* (α = 0.53), reflecting caregivers’ discomfort; *minimization reactions* (α = 0.79), reflecting caregivers’ devaluation of the child’s problem or emotions. The other three reflect positive reactions: *expressive encouragement* (α = 0.87), reflecting caregivers’ acceptance and promotion of the child’s negative emotional expressions; *emotion-focused reactions* (α = 0.87), reflecting strategies used to help the child feel better; and *problem-focused reactions* (α = 0.80), reflecting strategies used to help the child to solve the problem that caused distress.

Following Bost and colleagues [33], and excluding subscales with α < 0.60, two composites were generated: the *negative emotion regulation* (average of *punitive* and *minimization reactions* subscales; α = 0.86), and the *positive emotion regulation* (average of *expressive encouragement*, *emotion-focused reactions*, and *problem focused reactions* subscales; α = 0.91).

### 2.3. Procedures

Data collection was carried out in schools in Lisbon (26%) and online using Qualtrics during the 2021–2022 school year. We used convenience sampling. First, participants were presented with informed consent, informing the objectives of the study and anonymity. Additionally, before completing the questionnaires, they were asked to provide information regarding demographic data and the complementary feeding method previously adopted when the children were babies. This study was approved by the Ethics Committee (I/038/06/2020).

### 2.4. Analytic Plan

Before our main analyses, descriptive statistics were explored. Normality and homoscedasticity of the variances were tested. ANOVAs were used to test for significant differences between caregivers’ who previously adopted distinct complementary food methods regarding demographics and variables in the study (i.e., caregivers’ feeding practices and emotional regulation strategies). In the variables in which the assumption of homoscedasticity was not verified, the statistical analysis was performed using the Welch correction of ANOVA. Hierarchical regression analysis was also performed. All statistics were run using the Statistical Package for the Social Sciences (SPSS, Version 28.0, Armonk, NY, USA).

## 3. Results

Most of the participants (70.4%) reported using the TSF method to introduce complementary food, 16.8% said they used the BLW method and 12.8% used both methods simultaneously (mixed method). For the following analyses, two groups were created: a traditional (which included caregivers who used the TSF method) and a mixed group (which included caregivers who used BLW exclusively and those who used it simultaneously with the TSF). There was a significant difference in mothers’ age (*F*(175,1) = 23.39, *p* < 0.001; *M* = 37.84, *SD* = 5.03 for traditional and *M* = 34.02, *SD* = 4.14 for mixed) and in fathers’ age (*F*(179,1) = 9.10, *p* < 0.01; *M* = 39.65, *SD* = 5.68 for traditional and *M* = 36.73, *SD* = 6.03 for mixed), with older parents using the traditional method more often. Children in the traditional group spend more time at school compared to the mixed group (*F*welch = 62.26, *p* < 0.05; *M* = 7.57, *SD* = 1.10 for traditional and *M* = 6.90; *SD* = 2.15 for mixed). A significant difference was also found regarding firstborns (χ2(1,179) = 6.09, *p* < 0.01, φ = −0.18), with more firstborns than expected in the mixed group. No other differences were found.

### 3.1. Parental Feeding Practices Depending on the Complementary Feeding Method

As represented in Table 1, our results revealed significant differences in relation to *pressure* practices, depending on the method of complementary food introduction (*F*(1,178) = 5.00; *p* < 0.05). Specifically, parents who reported using a non-traditional complementary feeding method revealed using less *pressure to eat* (*M* = 2.43; *SD* = 0.98) compared to parents who used a TSF method (*M* = 2.80; *SD* = 0.97).

A hierarchical regression analysis was performed with demographics on the first block and adding the feeding method on the second block. The first block was not significant. Adding the feeding method improved the model (ΔR = 0.02; ΔF = 3.91, *p* < 0.05), and this was the only one with a significant coefficient (β = −0.16, *p* < 0.05).

### 3.2. Parental Responses to Children’s Negative Emotions Depending on the Complementary Feeding Method

Parents’ distress reactions were not included in the following analysis due to the poor alpha (α = 0.53). The results showed significant differences in *minimization reactions* depending on the complementary food introduction group (*F* = 8.49; *p* < 0.01). Specifically, parents who used the non-traditional method reported using fewer *minimization reactions* to children’s negative emotions (*M* = 2.49; *SD* = 0.87), compared to parents who used a non-traditional method (*M* = 2.95; *SD* = 0.86). Significant differences in emotion-focused reactions were also revealed (*F* = 4.75; *p* < 0.05). Specifically, parents who reported using a traditional complementary feeding method were found to have more problem-focused reactions (*M* = 6.12; *SD* = 0.77), compared with parents who used a non-traditional complementary feeding method (*M* = 5.76; *SD* = 1.08) (see Table 2).

## 4. Discussion

Our study aimed to explore if the prior implementation of different methods of complementary food introduction by parents was related to differences in their emotional and feeding responsiveness. Specifically, we intended to explore how different they may be in terms of the emotional regulation strategies and feeding practices they report using with their children during the preschool years. The results show that caregivers who followed different complementary feeding methods tend to be different at several levels, for instance, in terms of their demographic characteristics (e.g., age). This is in line with previous evidence showing that parents who follow these approaches may be different [2,15,16,22]. Our findings also suggest that these differences may continue to be observed later, during preschool years, expanding previous knowledge by showing some differences in the way caregivers respond to children in feeding contexts and when children become upset. 

Regarding feeding responsiveness, the results indicate that, compared with the caregivers who followed a traditional method, those who followed a BLW/mixed approach are less likely to pressure their preschool children to eat more foods or specific foods, ignoring their signals of hunger and satiety. This is in line with the results of Brown and Lee [30] and is consistent with a philosophy in which children should not be forced to eat, respecting their needs in terms of feeding time and what and how they want to eat. This reflects a more responsive eating style, which can be evident since parents decide how to introduce complementary foods to their children [1,9,23].

If evidence relating different complementary feeding methods with responsive feeding is scarce, it is even more difficult to locate data considering emotional responsiveness at this level. Concerning emotional responsiveness to children’s negative emotions, our findings reveal that, compared with the caregivers who followed a traditional method, caregivers who followed a non-traditional approach are less likely to minimize or devaluate the child’s negative emotional expression and state. On the other hand, caregivers who followed a traditional method are more likely to use a problem-focused strategy to deal with preschoolers’ negative emotions, by helping the child to solve the problem that caused him/her distress. These findings suggest that both caregivers that followed a more standard vs. a less standard weaning method may later be responsive to their preschoolers’ negative emotions, albeit they may do it in different ways. In this sense, it is possible that caregivers’ who followed a baby-led related approach can be responsive to their children’s negative emotions in a more emotionally supportive manner [23], whereas caregivers who followed a traditional method can be responsive to their children’s negative emotions in a more practical supportive manner.

Although these results are interesting and promising, they should be viewed with caution due to the nature and limitations of this study. This is a retrospective, comparative cross-sectional, exploratory study with a small sample size, which includes a limited number of participants who indicated they had followed the BLW method. Additionally, as in most studies, participants self-identify themselves as previously followed a specific complementary feeding method and were asked to estimate the frequency of its use and the proportion of food that they provided as purées or spoon-fed to their children when they were babies, with all the ambiguity this may imply [2,22]. Future studies should seek to overcome these limitations and will also benefit from a multi-informant (e.g., mothers and fathers), multi-method (e.g., self-reported and observational measures) and multi-context approach (e.g., family and schools). Specifically, longitudinal research is needed to disentangle associations between prior complementary feeding methods and later responsive parenting during preschool years.

It is plausible to speculate that parents who followed a BLW/mixed method are more likely to use responsive practices, but it is also possible that responsive parenting was the primary reason why they had implemented this complementary feeding method previously and why they continue to respond to their children during preschool years using more responsive practices. Thus, prospective data will help to understand if a particular complementary feeding method could encourage/discourage some responsive/nonresponsive feeding and emotion regulation strategies or whether these parenting practices make more/less sense and became more/less attractive to parents depending on their degree of responsiveness [22,23].

Additionally, it is possible that no single method, even BLW that appears to be more responsive in its nature (e.g., [22]), is feasible or suitable for all children at all times, and being a responsive caregiver is also being able to choose what fits better the child’s characteristics and needs. A child’s temperament, feeding or weight problems, and experience in choking are some infant characteristics highlighted as playing a role in parents’ decision about a feeding method. Additionally, caregivers’ own characteristics (e.g., maternal anxiety), knowledge and understanding about the advantages and disadvantages of a particular feeding method, as well as fears and concerns (frequently related to the possibility of chocking, nutrient deficits, allergic reactions and lower energy intakes) may condition these choices [2,14,17,22,23,40,41]. Considering all these factors, caregivers can even opt for a mixed approach to the detriment of an exclusively traditional or BLW approach, taking advantage of the potential that each of these methods can offer to a given child in certain circumstances. Most important is that caregivers’ choices reflect responsive parenting [23].

Regardless of the limitations of the present study, our findings contribute to the advancement of knowledge about the associations between complementary feeding methods and responsive parenting. The evidence suggests that emotional responsiveness and feeding responsiveness are linked facets of responsive parenting [31,33,34,35,36], and we know that responsive feeding is a major factor enhancing healthy eating behaviors and reducing the risk for weight problems from early in life [22,42]. Thus, data generated at this level are useful for the design of preventive practices to reduce childhood obesity risk and contribute positively to helping caregivers, through psychoeducation.

## Figures and Tables

**Table 1:** Differences in parental feeding practices by complementary food method.

		Method			
		Traditional	Mixed		
		*M* (*SD*)	*M* (*SD*)	*F*	*p*
CFPQ	emotion regulation	1.40 (0.51)	1.44 (0.50)		
	encourage balance and variety	4.65 (0.49)	4.76 (0.36)		
	environment	4.46 (0.66)	4.44 (0.56)		
	involvement	3.56 (1.02)	3.90 (1.02)		
	modelling	4.27 (0.74)	4.40 (0.70)		
	monitoring	4.46 (0.65)	4.40 (0.67)		
	pressure	2.80 (0.97)	2.43 (0.98)	5.00	0.03
	restriction for weight	2.14 (0.72)	2.03 (0.68)		
	pressuring feeding styles	2.14 (0.58)	1.95 (0.63)		
	healthy feeding styles	4.18 (0.50)	4.33 (0.44)		

*M* = mean; *SD* = standard deviation; *F* = asymptotic test; *p* = *p*-value; CFPQ = Comprehensive Feeding Practices Questionnaire.

**Table 2:** Differences in parental reactions to children’s negative emotions by complementary food method.

		Method			
		Traditional	Mixed		
		*M* (*SD*)	*M* (*SD*)	*F*	*p*
CCNES	punitive reactions	1.93 (0.71)	1.87 (0.74)		
	minimization reactions	2.95 (0.86)	2.49 (0.87)	8.49	0.004
	expressive encouragement	5.53 (0.95)	5.55 (1.13)		
	emotion-focused reactions	6.12 (0.77)	5.76 (1.08)	4.75	0.031
	problem-focused reactions	6.09 (0.70)	5.86 (1.09)		
	negative emotion regulation	5.91 (0.69)	5.73 (1.01)		
	positive emotion regulation	2.51 (0.57)	2.33 (0.62)		

*M* = mean; *SD* = standard deviation; *F* = asymptotic test; *p* = *p*-value; CCNES = Coping with Children’s Negative Emotions Scale.

## Data Availability

The raw data supporting the conclusions of this article will be made available by the authors, without undue reservation.
